# Reciprocal signaling between quorum sensing mutants: A model for division of labor.

**DOI:** 10.17912/micropub.biology.001326

**Published:** 2024-10-08

**Authors:** Farah Abdul-Rahman, Joao Xavier

**Affiliations:** 1 Computational and Systems Biology Program, Memorial Sloan Kettering Cancer Center, New York, United States

## Abstract

Division of labor, the specialization of subsets of individuals in complementary tasks, increases population efficiency and fitness. We explored swarming motility in *Pseudomonas aeruginosa* quorum sensing mutants as a model for studying the division of labor. Deletion of the signal synthesis genes *lasI* or *rhlI* disrupts swarming, but co-culturing *ΔlasI* and *ΔrhlI* restores it in a density-dependent manner. This indicates a successful division of labor where *ΔrhlI* produces the signal necessary for the *ΔlasI* mutant, and the *ΔlasI* reciprocates. We used RNA sequencing to identify additional genes potentially involved in division of labor. Our findings underscore *P. aeruginosa* swarming as a tractable bacterial model for the division of labor among cells—a hallmark of differentiated multicellularity.

**Figure 1. P. aeruginosa QS mutants exhibit density-dependent division of labor during swarming motility:**
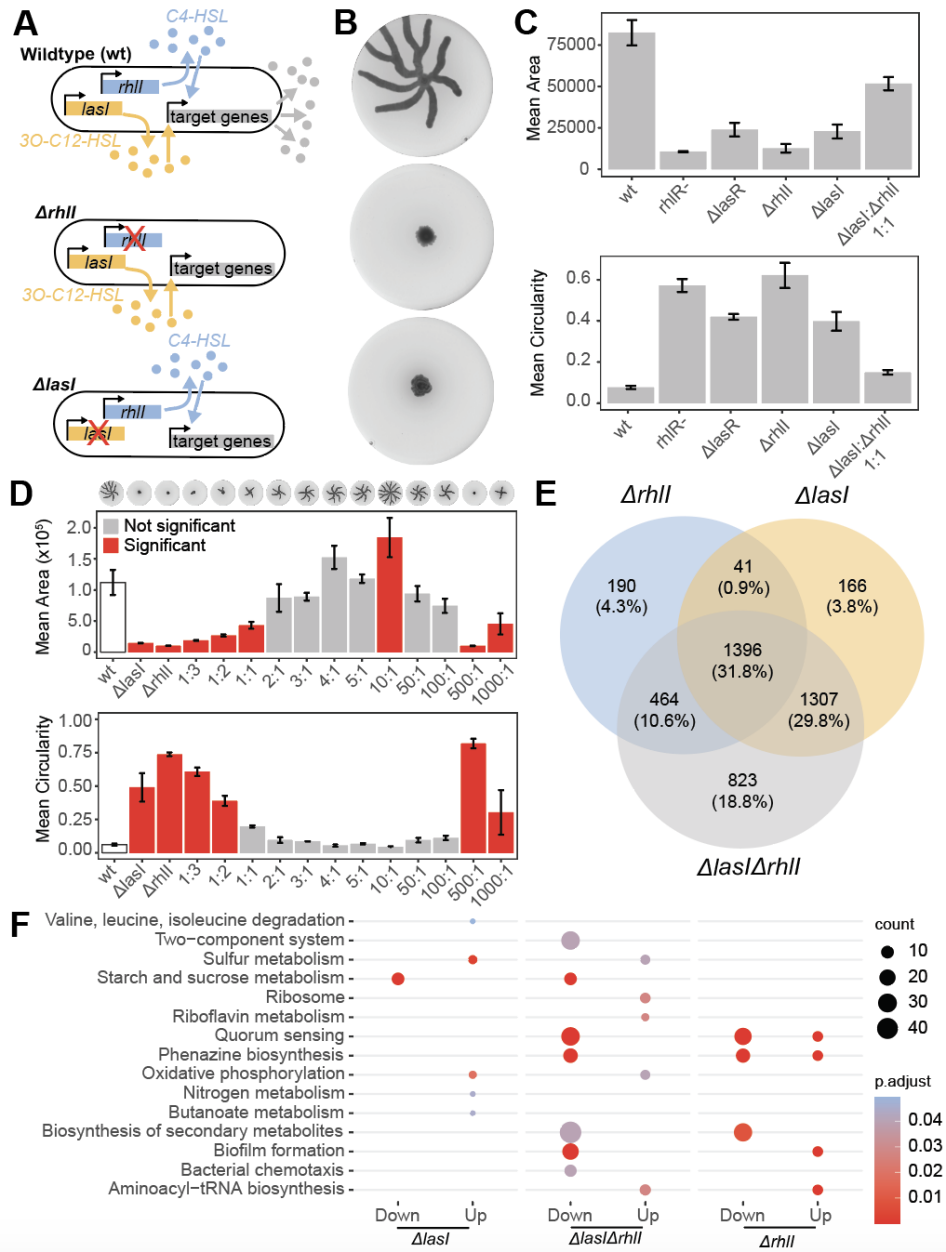
The two QS systems rhl and las regulate group-level genes. Knocking out either autoinducer synthase, *rhlI* and *lasI,* disrupts the signaling pathway (A). Swarming motility is a population-level phenotype that is lost in both *ΔrhlI* and *ΔlasI* monocultures (B). Co-culturing *ΔrhlI* and *ΔlasI* in a 1:1 ratio incompletely restores wildtype fitness (area (mm²)) and phenotype (circularity (0-1)) exhibiting potential for division of labor. Error bars represent the standard error of the mean (C). Co-culturing *ΔrhlI* and *ΔlasI* in a range of ratios restore wildtype (white) fitness and phenotype to varying degrees. Error bars represent the standard error of the mean. Dunnett's test was used to compare the co-culture swarms to wildtype and colonies with statistically significant differences are colored in red (D). Number of shared and distinct differentially expressed genes between QS mutants from RNAseq experiments (E). KEGG enrichment analysis for significantly up- and down-regulated genes across strains and the count of genes belonging to a set (F).

## Description

Division of labor is a task allocation process in which individuals within a group specialize in distinct tasks resulting in greater efficiency and increased population fitness (Dal Co et al., 2018; West & Cooper, 2016) . It is considered a prerequisite for complex biological interactions, such as those necessary for multicellularity (Kirk, 2005; West et al., 2015) . Recently, it has become increasingly clear that division of labor is a significant sociomicrobial phenomenon, playing a crucial role in structuring populations of free-living cells, including bacteria (Ackermann et al., 2008; Kim et al., 2016) . *Pseudomonas aeruginosa* , an opportunistic bacterium, is an excellent model for studying social interactions, such as cheating and cooperation, due to its extensive repertoire of secretions that function as public goods and are susceptible to exploitation (Armbruster et al., 2019; Guadarrama-Orozco et al., 2023; Monaco et al., 2022; Xavier et al., 2011; Yan et al., 2019) . Here, we leverage two of *P. aeruginosa* ’s best studied quorum sensing (QS) molecules, known to be essential for swarming motility, to explore its potential as a model for division of labor. We also perform RNAseq to generate a candidate list of relevant downstream genes.

In bacteria, QS systems allow cells to monitor population density and regulate the expression of group-level genes accordingly. A QS system typically has two components: an autoinducer synthase, which produces a diffusible signaling molecule called an autoinducer, and its cognate receptor, which detects the autoinducer and regulates the expression of downstream genes (Williams, 2007) . The las and rhl systems are two well-characterized QS systems in *P. aeruginosa* that regulate collective phenotypes, including swarming motility, which is a population-level movement that enables the colony to expand and multiply to higher numbers by harvesting nutrients across a wider area. The genes *lasI* and *rhlI* encode synthases which are involved in the production of the autoinducers N-3-oxo-dodecanoyl-L-Homoserine lactone (3O,C12-HSL) and N-butanoyl-L-homoserine lactone (C4-HSL) respectively (Daniels et al., 2004) . Deleting either *lasI* or *rhlI* prevents the production of their respective autoinducers and disrupts the signaling cascade that controls QS target genes ( **Figure 1A** ). Interestingly, QS gene mutants can act as cheats by benefiting from other group-level behaviors, such as siderophore production, without contributing to them (Diggle et al., 2007; Sandoz et al., 2007; Venturi et al., 2010) .

Swarming motility requires the synthesis and secretion of rhamnolipids, which reduce surface tension and facilitate the coordinated movement of bacterial cells across surfaces (Déziel et al., 2003) . When wildtype *P. aeruginosa* is spotted in the center of a swarming plate, the colony expands by forming bifurcating tendrils. In contrast—and as expected from the known signaling cascade—when *ΔlasI* and *ΔrhlI* were inoculated independently, swarming was significantly reduced or completely absent ( **Figure 1B** ). Given that swarming colonies expand on a 2-dimensional plane, we used colony area as a proxy measurement for population fitness. We also observed that colony circularity was inversely correlated with increased swarming, providing a simple phenotypic readout. Both *ΔlasI* and *ΔrhlI* mutants when inoculated independently exhibited reduced fitness compared to wildtype. We also tested two cognate receptor mutants *rhlR* -, a transposon mutant from (Liberati et al., 2006) , and *ΔlasR* , a deletion mutant. Both receptor mutants also demonstrated decreased fitness relative to the wildtype, consistent with the expectation that perturbations in autoinducer receptors disrupt the QS signaling cascade. Interestingly, although both las and rhl mutants exhibited severely compromised swarming ability, las mutants displayed a slightly better swarming capability ( **Figure 1C** ), consistent with previous observations (Köhler et al., 2000).

For division of labor to occur, two core conditions must be met 1) different individuals must carry out distinct and complementary tasks, and 2) individuals must be able to cooperate and demonstrate increased fitness when together compared to when alone (West & Cooper, 2016) . Since *ΔlasI* and *ΔrhlI* produce complementary autoinducers, we hypothesized that co-culturing *ΔlasI* and *ΔrhlI* could restore swarming through division of labor. To test this, we used an engineered synthetic system in which we co-cultured *ΔlasI* and *ΔrhlI* at a 1:1 ratio and found that population fitness increased relative to monocultures of either strain. Still, it did not fully restore fitness to wildtype levels. Mean circularity also decreased compared to monocultures, but remained slightly higher than wildtype levels ( **Figure 1C** ). This indicates that while the synthetic population with a 1:1 ratio of *ΔlasI* and *ΔrhlI* could divide labor, additional factors prevented swarming at the wildtype level.

We then investigated whether the ratio of co-cultured mutants influenced the outcome of the division of labor. We co-cultured *ΔlasI* and *ΔrhlI* strains at a range of ratios and found that increasing the ratio of *ΔlasI* to *ΔrhlI* decreased fitness and increased circularity, resembling the monoculture of either strain. In contrast, increasing the ratio of *ΔrhlI* to *ΔlasI* increased resemblance to wildtype fitness and circularity up to a ratio of 100:1 after which the trend reversed, and populations began to resemble monocultures again. Surprisingly, at a 10:1 ratio the mutant co-culture area was significantly greater than wildtype when we used a Dunnett’s test to compare them, suggesting that evolving division of labor in QS might even confer advantages over the wildtype population ( **Figure 1D** ).

To further support our observations, we evaluated the suitability of three mathematical models in describing the relationship between colony area and *ΔrhlI* frequency in co-cultures, all of which significantly fit our data. Among the quadratic (p = 0.0004), cubic (p < 0.0001), and spline (p < 0.0001) models, the spline model explained the highest proportion of variance (80.62%), followed by the cubic model (58.76%) and the quadratic model (35.01%) ( **Extended data** ). Therefore, we proceeded with the spline model to predict peak area as a proxy measurement for peak fitness, estimating a maximum value of 1.84 x 10⁵ mm² at a *ΔrhlI* frequency of ~0.9 (corresponding to a *ΔrhlI* : *ΔlasI* ratio of 10:1). As the mean area of the wildtype (1.1 x 10⁵ mm²) falls below the lower bound of the peak fitness estimate's confidence interval (95% CI: 1.58 x 10⁵ – 2.11 x 10⁵ mm²), we concluded that the *ΔrhlI:ΔlasI* ratio of 10:1 shows a significantly higher fitness compared to the wildtype.

QS systems regulate many target genes. To study the genes potentially involved in the division of labor, we performed RNAseq of wildtype, *ΔlasI* , *ΔrhlI* and *ΔrhlIΔlasI* strains in three different conditions: synthetic glucose media, synthetic glucose media without iron, and synthetic succinate media. Our rationale was that by sampling across three different environments, we could identify core genes differentially expressed independently of environmental influences, given the complex network and multi-directional feedback of QS systems (Wilder et al., 2011) . We found that *ΔlasI* shared 1,437 (32.7%) differentially expressed genes with *ΔrhlI* ( **Figure 1E** ). These shared genes were excluded as candidates for division of labor because they could not serve complementary functions ( **Extended data** ), thereby failing to meet the first requirement for division of labor mentioned above.

Kyoto Encyclopedia of Genes and Genomes (KEGG) enrichment analysis revealed that *ΔlasI* showed differential expression in sulfur, starch, nitrogen and butanoate metabolism genes while *ΔrhlI* showed differential expression in QS, biosynthesis and biofilm formation genes. Although many functional categories were shared between the single-gene knockouts, *ΔlasI* and *ΔrhlI* , we identified several categories unique to the simultaneous disruption of both genes. These unique categories included genes involved in ribosome function, riboflavin metabolism, and chemotaxis ( **Figure 1F** ).

In this study, we demonstrated that QS mutants can exhibit division of labor during *P. aeruginosa* swarming. Using synthetic populations composed of *ΔlasI* and *ΔrhlI* at equal ratios, we observed a partial but effective division of labor, however, the degree to which this observation can be extended to natural contexts remains unknown. Excitingly, this behavior was density-dependent with the surprising finding that a 10:1 ratio of *ΔrhlI* to *ΔlasI* , significantly enhanced population fitness, surpassing that of the wildtype. Using RNAseq, we identified a broader list of candidate genes for division of labor and applied KEGG enrichment to determine functional categories to which they belong. This approach allowed us to compile a comprehensive list of differentially expressed genes and the corresponding strains in which they were identified ( **Extended data** ). Our results contribute to the growing body of literature on using engineered systems to test the capacity for the evolution of division of labor (Mridha and Kümmerli 2022) .

Taken together, our results raise an important question, which is whether cheating, considered a trait that benefits the individual at the cost of the population, might serve as an evolutionary intermediate step to higher complexity cooperation like division of labor. QS autoinducer mutants can act as cheats when mixed with wildtype *P. aeruginosa* (Mund et al., 2017) , but in our study we found that when autoinducer mutants in complementary QS systems are mixed with each other, this leads to synergy in which population fitness was comparable to wildtype, and in some cases even higher than wildtype fitness. This highlights the possibility that division of labor could, in some cases, occur when two subpopulations of cells that typically cheat in the presence of wildtype instead cooperate with each other to provide complementary public goods.

Our findings underscore that *P. aeruginosa* and its QS genes are an excellent model for exploring the dynamics of division of labor. This study not only advances our understanding of sociomicrobial interactions but also provides a valuable resource for further investigations into the molecular mechanisms underlying the observed phenotypic changes.

## Methods

**Strains and swarming assay**

*P. aeruginosa* strains used in this study are listed in **Table 1** . Swarming plates were prepared with 200mL of 5X minimal salts stock solution, 1mL of 1M MgSO4, 100μL of 1M CaCl _2_ , 25mL of 200g/L casamino acids solution, 0.5% agar and milliQ water up to 1L. The 5X minimal salts stock solution was prepared with 64g Na _2_ HPO _4_ 𑇐7H _2_ O, 15g of KH _2_ PO _4_ , 2.5g of NaCl and milliQ water up to 1L. Strains were grown in 3mL LB overnight cultures at 37°C and 1mL of each culture was washed twice with phosphate-buffered saline (PBS). Samples were back-diluted to OD600 0.1 and 2μL were spotted in the center of the swarming plates and incubated at 37°C for 24 hours.

**Swarming image collection and analysis**

Swarming plates were imaged using a GelCount ^TM^ . ImageJ was used to quantify swarm area (mm²) and swarm circularity (0-1).

**Growth conditions used for RNAseq**

The four strains wildtype, *ΔrhlI* , *ΔlasI* and *ΔrhlIΔlasI* were grown in three media types. 1) Synthetic glucose media was prepared with 200mL of 5x Minimal Salts, 1mL of 1M MgSO4, 100μL of 1M CaCl _2_ , 14.4mL of 1.25M (NH4)2SO4, 15 mL of 50% Glucose, 40μL of 0.23M FeSO _4_ and milliQ water up to 1L. 2) Synthetic glucose media without iron was prepared as described above with the iron substituted with the same volume of milliQ H2O. 3) Succinate media was prepared with 6g of K _2_ HPO _4_ , 3g of KH _2_ PO _4_ , 1g of (NH _4_ ) _2_ SO _4_ , 0.2g of MgSO _4_ ·7H _2_ O, 4g succinic acid, 1.1g of NaOH and milliQ water up to 1L.

**RNAseq and analysis**

2mL of culture was spun down for 30s, supernatant discarded, cell pellets flash frozen and stored at -80°C. Frozen cell pellets were submitted on dry ice to Azenta for extraction, library generation, sequencing and count generation. Downstream analysis was analyzed in-house using custom R scripts. Outlier samples that were in disagreement with other replicates were filtered out. Counts were normalized and differentially expressed genes were called using DESeq2 (Love et al., 2014) . KEGG Enrichment Analysis was performed using clusterProfiler (Yu et al., 2012) . Sequencing reads are deposited in the NCBI Sequence Read Archive (SRA) under BioProject PRJNA1144160.

## Reagents

**Table:** 

**Strain**	**Genotype**	**Available from**
PA14 *∆rhlI*	Pseudomonas aeruginosa	Debra Hogan (DH169)
PA14 *∆lasI*	Pseudomonas aeruginosa	Debra Hogan (DH132)
PA14 *∆lasI ∆rhlI*	Pseudomonas aeruginosa	Debra Hogan (DH242)
PA14 *rhlR-*	Pseudomonas aeruginosa	Ausubel Lab
PA14 *∆lasR*	Pseudomonas aeruginosa	Debra Hogan (DH164)
PA14 *∆rhlI* attTn7::PA1/04/03-gfp(ASV)	Pseudomonas aeruginosa	This study

## Extended Data

Description: Table of genes, log2 fold change, p-value, and which strains they were differentially expressed in.. Resource Type: Dataset. DOI: 10.22002/5cz4w-8az98

Description: Figure of three models fitted to area vs. frequency of ΔrhlI. Blue line shows fit and shaded area shows 95% confidence intervals.. Resource Type: Image. DOI: 10.22002/jq7dr-0yc95
